# On the cicada genus *Nipponosemia* Kato, with description of one new species from China (Hemiptera, Cicadidae)

**DOI:** 10.3897/zookeys.293.4649

**Published:** 2013-04-19

**Authors:** Mingsheng Yang, Cong Wei

**Affiliations:** 1 Key Laboratory of Plant Protection Resources and Pest Management of Ministry of Education, Entomological Museum, Northwest A&F University, Yangling, Shaanxi 712100, China

**Keywords:** Cicadatrini, Cicadinae, Cicadettinae, morphology, taxonomy, biogeography

## Abstract

The cicada genus *Nipponosemia* Kato is reviewed. Four species are illustrated, photographed and described, including three known species and one new species. A key to all species of this genusis presented, and information on the biology of *Nipponosemia* are provided. The systematic status of the tribe Cicadatrini and biogeography of *Nipponosemia* are discussed.

## Introduction

The cicada genus *Nipponosemia* was established by Kato (1925a) based on external morphology, e.g., head (including eyes) about as wide as base of mesonotum, a little shorter than pronotum, mesonotum distinctly longer than pronotum, abdomen moderately robust and shorter than distance from head to cruciform elevation, timbal cover large, covering timbal almost entirely, opercula reaching middle of abdomen, fore wing with eight apical cells, hind wing with six apical cells, *etc*. This genus was formerly treated as a member of Cicadini (Metcalf 1963; Duffels and van der Laan 1985; Chou et al. 1997). However, Hayashi (1974) defined *Nipponosemia* as a member of Moganniini Distant (*sensu* Lee and Hayashi 2004, Pham and Yang 2009) after examining the male genitalia of *Nipponosemia* and its relatives. Chou et al. (1993) described two species from Guangxi Province of China for *Nipponosemia*, increasing the known species from two to four of this genus. Recently, Lee and Hill (2010) recognized Moganniini as a junior synonym of Cicadatrini Distant, and redefined the latter by including *Nipponosemia* Kato, *Cicadatra* Kolenati, *Psalmocharias* Kirkaldy, *Mogannia* Amyot and Serville and *Emathia* Stål. Wei et al. (2010) established another genus and species, *Shaoshia zhangi* Wei, Ahmed and Rizvi for the Cicadatrini. More recently, the genus *Klapperichicen* Jacobi was also included in the Cicadatrini by Lee (2012).

In the present paper we review the *Nipponosemia* including the description of one new species. A key to all the five species of *Nipponosemia* is provided. In addition, the biogeography of *Nipponosemia* and the systematic status of the tribe Cicadatrini are discussed.

## Material and methods

This study is based on specimens deposited in the Entomological Museum, Northwest A&F University, Yangling, China (abbreviated as NWAF in the text). The type specimens of the new species are also deposited in NWAF.

External morphology was observed and illustrated using a Motic SMZ 168-BL microscope. Photos were taken using a Scientific Digital micrography system equipped with an Auto-montage imaging system and a QIMAGING Retiga 4000R digital camera (CCD). The male genitalia were studied and illustrated using a compound light microscope (Nikon Eclipse 50i).

Terminology for morphological features follows that of Moulds (2005).

## Taxonomy

### Family Cicadidae Latreille Subfamily Cicadinae Latreille Tribe Cicadatrini Distant

#### Genus Nipponosemia

Kato, 1925

http://species-id.net/wiki/Nipponosemia

Nipponosemia Kato, 1925a: 55. Type species: *Abroma terminalis* Matsumura, 1913.

##### Diagnosis.

Body medium-sized. Head short, slightly produced anteriorly, not longer than pronotum; about as wide as base of mesonotum; postclypeus moderately swollen, longitudinally sulcate medially. Pronotum nearly trapezoid in dorsal view, wider than head; anterolateral margin not dentate, lateral angle of pronotal collar ampliated. Abdomen moderately obconical, usually shorter than distance from head to cruciform elevation; timbal cover somewhat semicircular, slightly wider than long, covering timbal almost entirely. Fore wing and hind wing with eight and six apical cells, respectively. Male pygofer with basal lobe absent; upper lobe present; uncus short, not dominant, median lobe of uncus weakly developed; claspers separated from each other in ventral view, with median clasper process long and lateral clasper lobe rounded; aedeagus cylindrical, long and somewhat stout, with six to eight spine-like processes apically and subapically.

##### Key to the males of the species of *Nipponosemia* Kato

**Table:** 

1	Fore wing with infuscations on most apical cells	2
–	Fore wing without infuscations on apical cells, or merely with a infuscation on apical cell 1	3
2	Body small (approximately 20mm in length); mesonotum with two pair of obconical marks originated from anterior margin; primary spine of fore femur slanted	*Nipponosemia metulata*
–	Body large (approximately 28mm in length); mesonotum with only one pair of obconical marks originated from anterior margin; primary spine of fore femur prostrate	*Nipponosemia guangxiensis*
3	Fore wing with an infuscation on apical cell 1	*Nipponosemia terminalis*
–	Fore wing without infuscations on apical cells	4
4	Pronotum with a pair of large reddish brown to dark brown patches with border black; male opercula with subapical portion enlarged toward body center, posterior margin broadly rounded	*Nipponosemia longidactyla* sp. n.
–	Pronotum without distinct markings; male opercula with apical two-thirds somewhat oblong, posterior margin strongly convex	*Nipponosemia virescens*

#### Nipponosemia terminalis

(Matsumura, 1913)

http://species-id.net/wiki/Nipponosemia_terminalis

Figures 1 3

Abroma terminalis Matsumura, 1913: 82.
Cicada fuscoplaga Schumacher, 1915: 109; Kato 1925b: 9.
Lemuriana terminalis , Matsumura 1917: 208.
Cicada terminalis , Kato 1925b: 9.
Nipponosemia terminalis , Kato 1925a: 56; Duffels and van der Laan 1985: 164; Chou et al. 1997: 123; Lee and Hayashi 2004: 61; Hayashi and Saisho 2011: 175.

##### Material examined.

1♂ (NWAF), China: Sichuan Prov., Chengdu, ?-VI-1951, coll. Huang Keren; 1♂ (NWAF), China: Sichuan Prov., Mt. Emeishan, 17-VII-1957, coll. Zheng Leyi and Cheng Hanhua; 1♀ (NWAF), China: Fujian Prov., Mt. Baiyunshan, 25-V-1987, coll. unknown; 1♂ (NWAF), China: Chongqing, Xiema, 25-VII-2007, coll. Wu Yiling; 1♂ (NWAF), China: Sichuan Prov., Mt. Emeishan, 7-VII-2010, coll. Wang Junchao.

##### Additional material.

1♀ (NWAF), China: Fujian Prov., Mt. Baiyunshan, 25-V-1987, coll. unknown.

##### Description.

**Figure 1.**
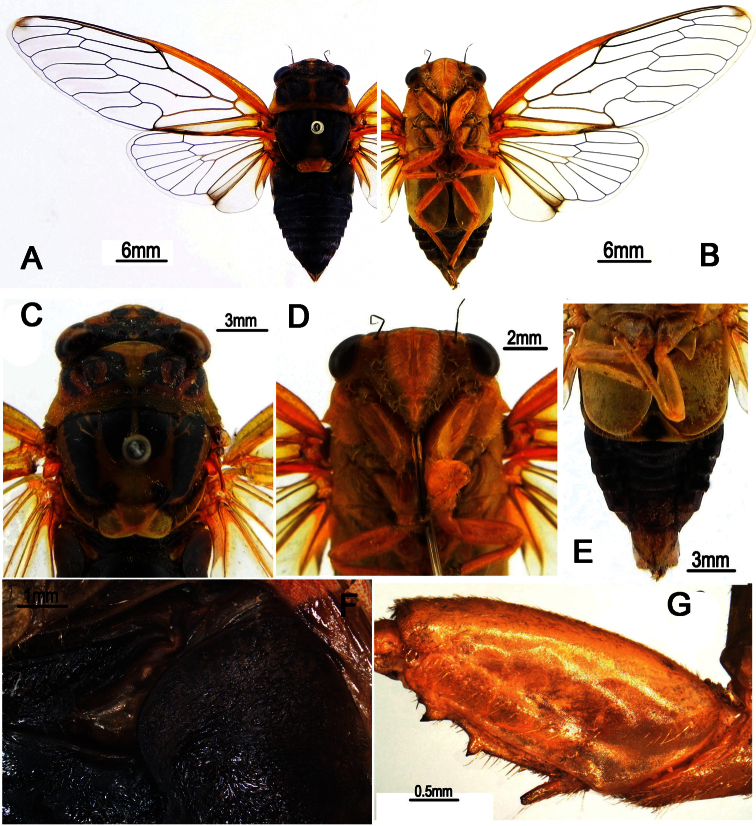
*Nipponosemia terminalis* (Matsumura, 1913),male. **A** habitus, dorsal view **B** habitus, ventral view **C** head and thorax, dorsal view **D** face **E** abdomen and posterior part of thorax, ventral view **F** timbal and timbal cover, dorsal view **G** left fore leg, showing the spines on fore femur.

Head (Fig. 1A–D) mostly yellowish brown, with black markings on vertex and postclypeus in dorsal view; clypeus brownish yellow and depressed; ocellus reddish, eye dark castaneous, distance between lateral ocellus and corresponding eye a little longer than distance between lateral ocelli; gena and lorum brownish yellow, with tuft of golden hairs; rostrum yellowish with apical half black, extending to apex of mid coxae.

Pronotum (Fig. 1C) with central longitudinal greenish yellow fascia well broadened at anterior part; symmetrically with two brown and black areas lateral to the central fasciae; pronotal collar greenish yellow. Mesonotum (Fig. 1C) mostly reddish yellow, with central longitudinal yellowish fascia extending to cruciform elevation; pair of somewhat obconical black fasciae lateral to the central longitudinal fascia short and curved outwardly, reaching to about 2/5 of mesonotum; pair of somewhat obconical black fasciae lateral to the short fasciae long and curved outwardly, with apices connecting with the black roundish spots enclosing scutal depressions; cruciform elevation greenish yellow. Ventral surface of thorax brownish yellow.

Legs (Fig. 1G) brownish yellow except for black pretarsal claws; fore femur with primary spine long, digitate and slanted; secondary spine short, sharp and erect; subapical spine short, sharp and slanted.

Wings (Fig. 1A–B) hyaline, veins in basal half yellowish brown and dark brown apically; fore wing with a light brown infuscation on apical part of apical cell 1.

Male abdomen (Fig. 1A–B) mostly black dorsally and yellowish green ventrally, with yellowish brown band on each posterior margin of terga 3–8; timbal cover (Fig. 1F) dark reddish brown; operculum (Fig. 1E) pale greenish yellow, extending slightly beyond posterior margin of abdominal sternite II, widest at half-length, medial margin somewhat convex, posterior margin rounded, lateral margin very weakly sinuate and gradually curved inwardly, medial margins nearly touching each other. Female abdomen mostly black dorsally and yellowish brown ventrally; operculum small, somewhat semicircular, with posterior margin extending not beyond posterior margin of abdominal sternite II, both opercula well separated from each other.

**Figure 2.**
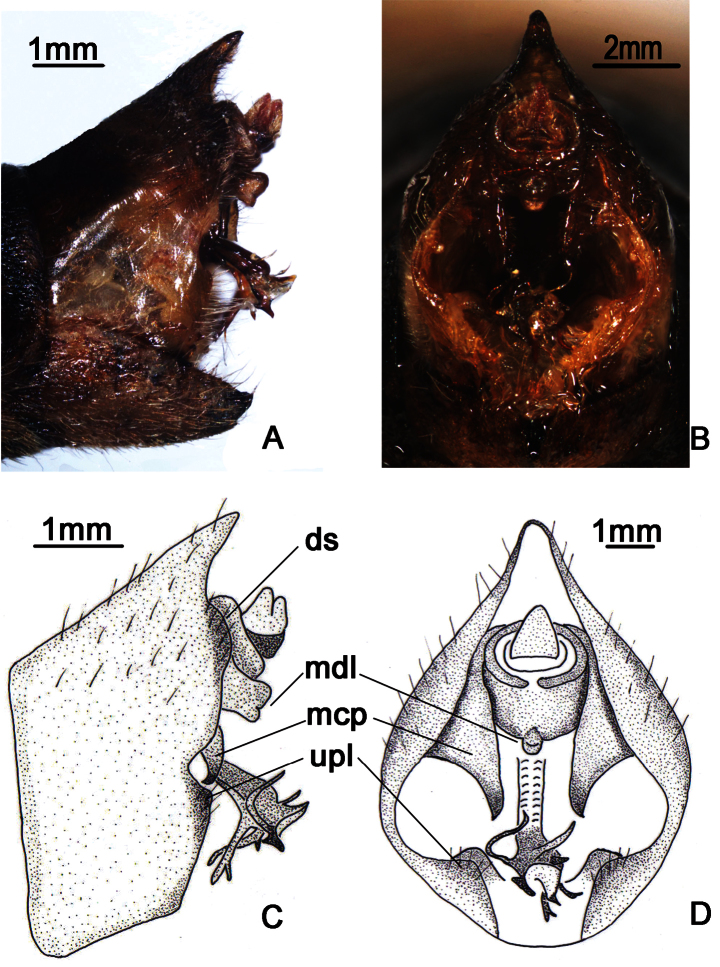
*Nipponosemia terminalis* (Matsumura, 1913),male. **A, C** male genitalia, left lateral view **B, D** male genitalia, ventral view. ds, distal shoulder; mcp, median clasper process; mdl, median lobe of uncus; upl, upper lobe of pygofer.

Male genitalia (Fig. 2A–D). Pygofer oval in ventral view; dorsal beak long, slightly protruding upwards in lateral view; distal shoulder very broad and sinuate, with somewhat triangular process near upper lobe of pygofer; upper lobe of pygofer short and obtuse in lateral view. Uncus with median lobe with rounded process adjacent anal tube in lateral view. Clasper in ventral view with median clasper process fairly broadened basally and narrowed apically, with apex acute and curved inwardly; lateral clasper lobe roundly developed, without distinct concave between median clasper process and corresponding lateral clasper lobe. Aedeagus with broadened and curved membranous sheet apically; eight short to long processes present on the sheet marginally, of which two long ones curved dorsad and the others curved downward in ventral view, with the basal-most ventral one the longest in lateral view. Posterior margin of sternite VII short and angularly produced.

**Figure 3.**
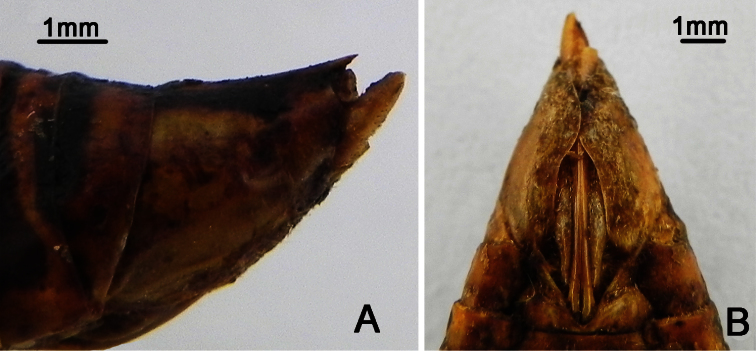
*Nipponosemia terminalis* (Matsumura, 1913),female. **A** female genitalia, left lateral view **B **female genitalia, ventral view.

Female pygofer (Fig. 3A–B) with dorsal beak short and acute, much shorter than protruding part of ovipositor; posterior margin of sternite VII with median incision very deep and broad, deep to about 4/5 the length of sternite VII.

**Measurements (**4♂♂, 1♀) (in mm). Body length: ♂ 25.0–26.0, ♀ 24.5; fore wing length: ♂ 27.0–30.0, ♀ 29.5; fore wing width: ♂ 9.5–10.5, ♀ 10.5; width of head including eyes: ♂ 7.5–9.5, ♀ 7.5; pronotum width (including pronotal collar): ♂ 9.0–10.0, ♀ 9.5; mesonotum width: ♂ 7.5–8.5, ♀ 8.0.

##### Biology.

This species is distributed from lowlands to low mountainous areas. Adults appear from May to August. They usually perch on low branches or trunks of various trees and sing in the sunshine (Lee and Hayashi 2004).

##### Distribution.

China (Sichuan, Fujian, Chongqing, Taiwan), Japan.

##### Remarks.

Hayashi and Saisho (2011) recorded the variation in body coloration of this species among geographic populations from the Ryukyus. The materials of this species from China mainland examined in the present paper are most externally similar to those distributed in Miyuan, Ishigaki Island. In addition, we include one female specimen as additional material for this species based on its external morphology and collecting data. This specimen has some differences with the one female specimen of *Nipponosemia terminalis* we examined in body coloration and the length of ovipositor. The identity of this female specimen needs to be investigated further when more specimens become available.

#### Nipponosemia metulata

Chou & Lei, 1993

http://species-id.net/wiki/Nipponosemia_metulata

Figures 4 6

Nipponosemia metulata Chou & Lei, 1993: 83; Chou et al. 1997: 125.

##### Material examined.

1♀ (NWAF), China: Guangxi Prov., Longzhou, 17-VI-1980, coll. Xi Fusheng; 1♂ (NWAF), China: Guangxi Prov., Longzhou, light strap, 21-V-1982, coll. unkuown; 1♂ (NWAF), China: Guangxi Prov., Ningming, light strap, 16-V-1984, coll. Zhi Tian.

##### Description.

**Figure 4.**
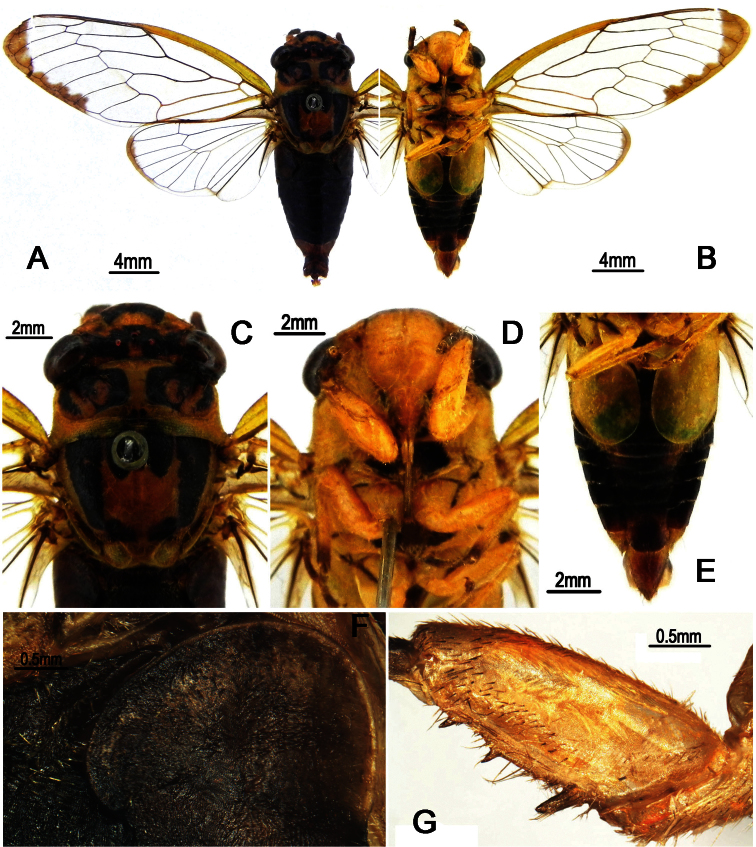
*Nipponosemia metulata* Chou & Lei, 1993, male. **A** habitus, dorsal view **B** habitus, ventral view **C** head and thorax, dorsal view **D** face **E** abdomen and posterior part of thorax, ventral view **F** timbal and timbal cover, dorsal view **G** left fore leg, showing the spines on fore femur.

Head (Fig. 4A–D) mostly brownish yellow, with black markings on vertex and postclypeus in dorsal view; clypeus brownish yellow and depressed; ocellus reddish, eye dark castaneous, distance between lateral ocellus and corresponding eye as long as distance between lateral ocelli; gena and lorum brownish yellow, with tuft of golden hairs; rostrum yellowish with apical half brown, extending to apex of mid coxae.

Pronotum (Fig. 4C) with central longitudinal brownish yellow fascia well broadened at anterior part; symmetrically with two brown and black areas lateral to the central fasciae; pronotal collar mostly greenish yellow. Mesonotum (Fig. 4C) mostly reddish yellow, centrally with pair of obconical black fasciae short and slightly curved outwardly, reaching to about 1/4 of mesonotum; pair of obconical black fasciae lateral to the short outwardly curved fasciae long and curved inwardly, with apices connecting with the black roundish spots enclosing scutal depressions; cruciform elevation mostly greenish yellow. Ventral surface of thorax mostly brownish yellow.

Legs (Fig. 4G) mostly yellow; tarsi and tibiae brown to dark brown and apices of pretarsal claws reddish brown; fore femur with primary spine long, digitate and slanted; secondary and subapical spines short, sharp and somewhat erect.

Wings (Fig. 4A–B) hyaline, veins generally in basal half yellowish green and dark brown apically; fore wing with continuous brown fascia along apical parts of apical cells 1–7 and associated outer margin of fore wing; hind wing with continuous brown fascia along apical parts of apical 1–4 and associated outer margin of hind wing.

Male abdomen (Fig. 4A–B) mostly black except for reddish brown tergite 8; timbal cover (Fig. 4F) dark brown; operculum (Fig. 4E) mostly brownish yellow with apical 1/3 mostly greenish, extending slightly beyond posterior margin of abdominal sternite II, obliquely ellipsoidal, subapical portion enlarged toward body center, posterior margin rounded, medial margins not touching each other. Female abdomen mostly black dorsally and reddish brown ventrally, with golden hairs on each posterior margin of terga 2–8; operculum small, somewhat semicircular, with posterior margin truncated, extending not beyond posterior margin of abdominal sternite II, both opercula well separated from each other.

**Figure 5.**
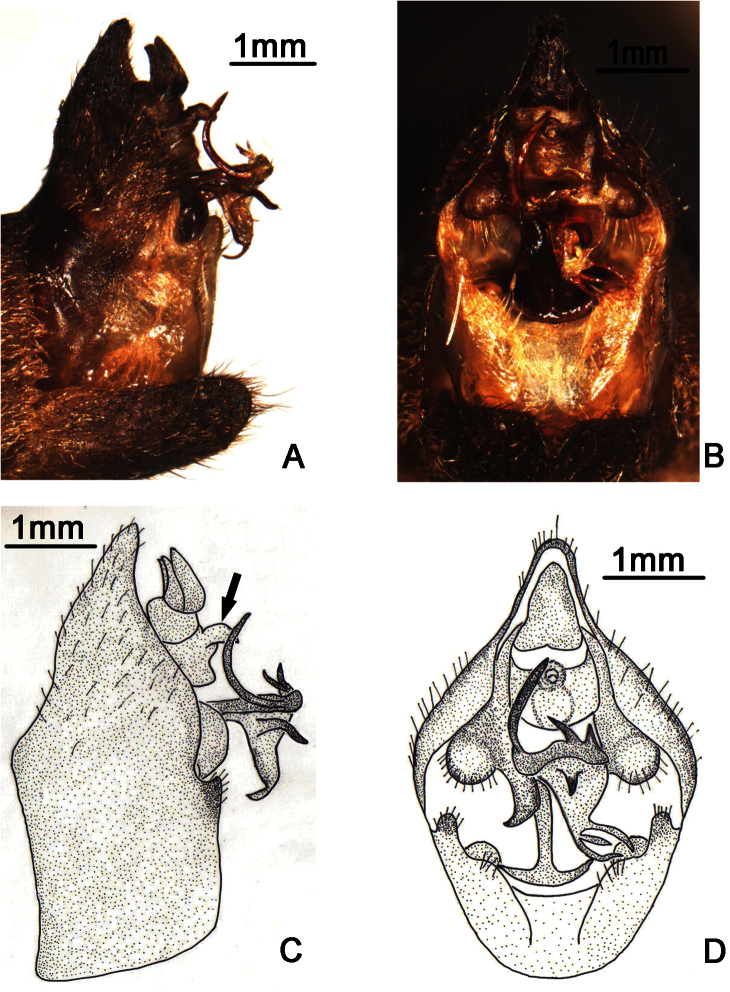
*Nipponosemia metulata* Chou & Lei, 1993, male. **A, C** male genitalia, left lateral view **B, D** male genitalia, ventral view.

Male genitalia (Fig. 5A–D). Pygofer oval in ventral view; dorsal beak well developed, protruding upwards in lateral view; distal shoulder very broadly rounded; upper lobe of pygofer produced posteriorly, triangular-shaped in lateral view. Uncus with beak-like process adjacent to anal tube in lateral view (as arrow indicated in Fig. 5C). Clasper in ventral view with median clasper process long, with apex acute and curved laterally, falcate in shape; lateral clasper lobe roundly developed. Aedeagus in ventral view with broadened membranous sheet apically, which is remarkably developed ventrally and bears seven short to long processes: one long and two short processes at the upper margin curved upward, three at the lower margin curved downward, and one short process arising medially in ventral view. Posterior margin of sternite VII short and rounded.

**Figure 6.**
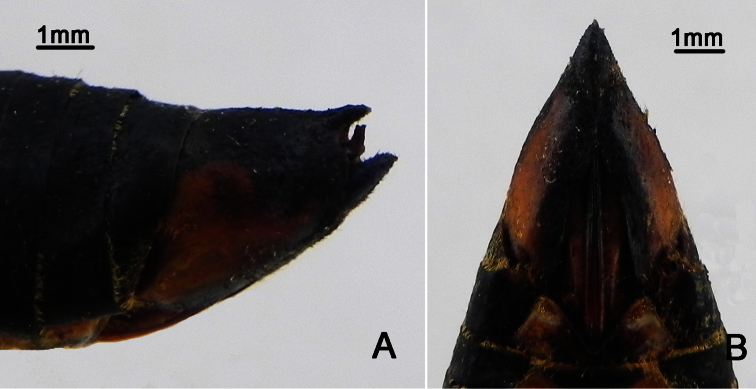
*Nipponosemia metulata* Chou & Lei, 1993, female. **A** female genitalia, left lateral view **B **female genitalia, ventral view.

Female pygofer (Fig. 6A–B) with dorsal beak short and acute, slightly shorter than protruding part of ovipositor; posterior margin of sternite VII with median incision broad and deep, deep to about 4/5 the length of sternite VII.

**Measurements (**2♂♂, 1♀**)** (in mm). Body length: ♂ 20.0–20.5, ♀ 22.0; fore wing length: ♂ 22.0–23.0, ♀ 24.5; fore wing width: ♂ 8.0–8.5, ♀ 9.0; width of head including eyes: ♂ 7.0–7.5, ♀ 8.5; pronotum width (including pronotal collar): ♂ 7.0–7.5, ♀ 8.5; mesonotum width: ♂ 6.0–6.5, ♀ 7.5.

##### Biology.

Unknown.

##### Distribution.

China (Guangxi).

##### Remarks.

This species is similar to *Nipponosemia terminalis*, but it can be distinguished from the latter by a smaller body size, the big markings on wings, the shape of clasper and aedeagus, and the shorter protruding part of ovipositor (Chou et al. 1993, 1997).

#### Nipponosemia guangxiensis

Chou & Wang, 1993

http://species-id.net/wiki/Nipponosemia_guangxiensis

Figures 7 9

Nipponosemia guangxiensis Chou & Wang, 1993: 84; Chou et al. 1997: 127; Pham and Yang 2009: 5.

##### Material examined.

1♂ (NWAF), China: Guangxi Prov., Ningming, 19-V-1984, coll. Wang Jijian; 1♀ (NWAF), China: Guangxi Prov., Longzhou, 17-V-1983, coll. Liu Sikong.

##### Description.

**Figure 7.**
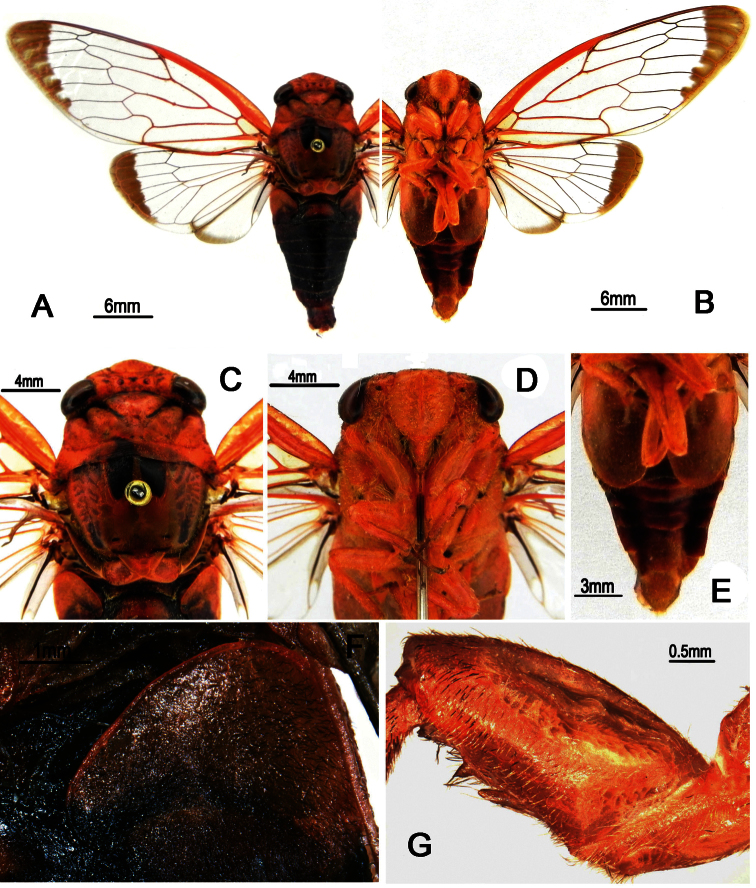
*Nipponosemia guangxiensis* Chou & Wang, 1993, male. **A** habitus, dorsal view **B** habitus, ventral view **C** head and thorax, dorsal view **D** face **E** abdomen and posterior part of thorax, ventral view **F **timbal and timbal cover, dorsal view **G** left fore leg, showing the spines on fore femur.

Head (Fig. 7A–D) mostly reddish brown, symmetrically with small black stripes on vertex; clypeus reddish brown and depressed; ocellous reddish, eye castaneous, distance between lateral ocellus and corresponding eye as long as distance between lateral ocelli; gena and lorum mostly reddish brown, with tuft of golden hairs and lorum symmetrically with pair of black stipes; rostrum reddish with apical half dark brown, extending to apex of mid coxae.

Pronotum (Fig. 7C) reddish brown, with paramedian and lateral fissures ochreous. Mesonotum (Fig. 7C) mostly reddish brown, centrally with pair of short and slightly outwardly curved obconical black fasciae, reaching to about the 1/4 of mesonotum; pair of slender short black stripes and irregular markings lateral to the outwardly curved fasciae; cruciform elevation reddish brown. Ventral surface of thorax mostly reddish brown.

Legs (Fig. 7G) mostly reddish brown; tarsi and pretarsal claws dark reddish brown; fore femur with primary spine long, digitate and prostrate; secondary spine short, sharp and erect; subapical spine short, sharp and almost prostrate.

Wings (Fig. 7A–B) hyaline, veins generally in basal half reddish brown and brown apically; fore wing with continuous brown fascia along apical parts of apical cells 1–7 and associated outer margin; hind wing with two continuous brown fascia: one along apical parts of apical cells 1–5 and associated outer margin, the other along outer margin of vannal region.

Male abdomen (Fig. 7A–B) mostly black dorsally and reddish brown ventrally, with central trapezoid reddish brown mark on tergite II; timbal cover (Fig. 7F) reddish brown; operculum (Fig. 7E) reddish brown, extending slightly beyond posterior margin of abdominal sternite II, widest at half-length, medial margin of operculum slightly convex, posterior margin rounded, lateral margin weakly sinuate and gradually curved inwardly, medial margins nearly touching each other. Female abdomen mostly black dorsally and yellowish brown ventrally, with golden hairs on each posterior margin of terga 2–8; operculum small, triangular, with posterior margin truncated, extending not beyond posterior margin of abdominal sternite II, both opercula well separated from each other.

**Figure 8.**
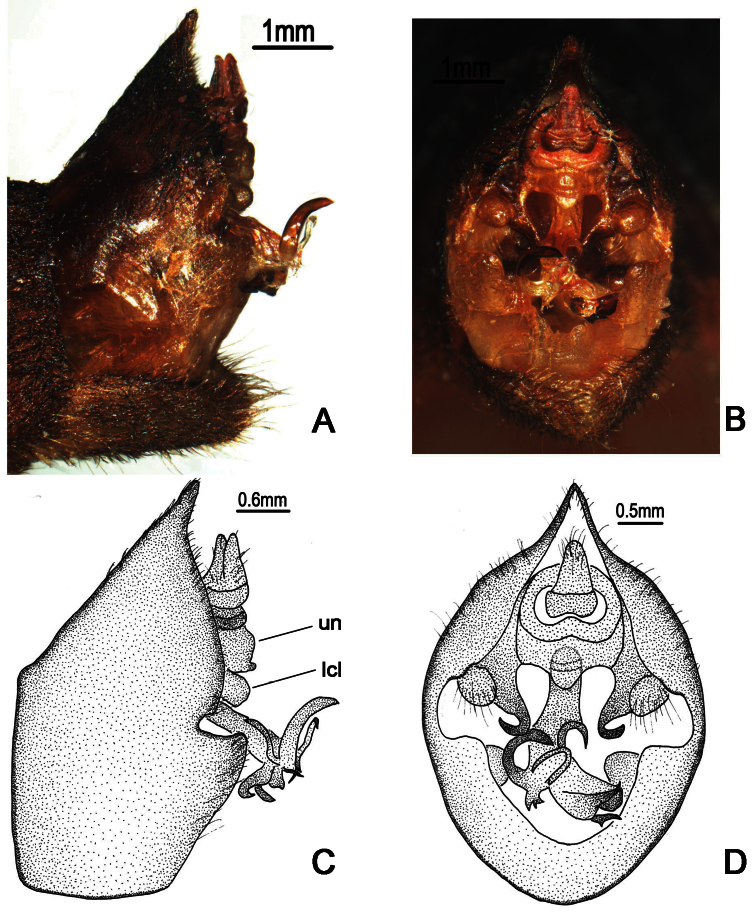
*Nipponosemia guangxiensis* Chou & Wang, 1993, male. **A, C** male genitalia, left lateral view **B, D** male genitalia, ventral view. un, uncus; lcl, lateral clasper lobe.

Male genitalia (Fig. 8A–D). Pygofer oval in ventral view; dorsal beak protruding upwards in lateral view; distal shoulder broadly rounded; upper lobe remarkably developed, forming a very large triangular-shaped protrusion in lateral view. Uncus with apex of median lobe slightly developed ventrally, forming a small process in both lateral and ventral views. Clasper in ventral view with median clasper process very long, broaden basally, apex strongly curved laterally, hook-like in shape; lateral clasper lobe roundly developed. Aedeagus in ventral view with three long apical processes curved dorsad and other three small to large lobe-like processes curved ventrad; the shortest lobe-like process with three short spines apically in ventral view; the medial lobe-like process bifurcate subapically, with apices acute; the third lobe-like process large, with apex somewhat rounded. Posterior margin of sternite VII short and rounded.

**Figure 9.**
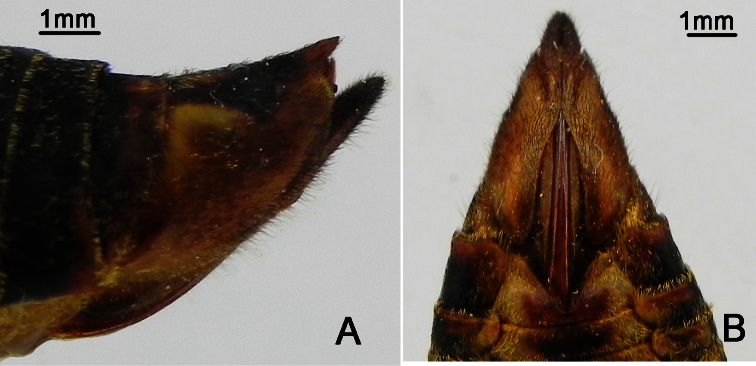
*Nipponosemia guangxiensis* Chou & Wang, 1993, female. **A** female genitalia, left lateral view **B** female genitalia, ventral view.

Female pygofer (Fig. 9A–B) with dorsal beak short and acute, shorter than protruding part of ovipositor; posterior margin of sternite VII with median incision large and broad, deep to about 4/5 the length of sternite VII.

**Measurements** (1♂, 1♀) (in mm). Body length: ♂ 28.0, ♀ 26.0; fore wing length: ♂ 31.0, ♀ 31.0; fore wing width: ♂ 11.5, ♀ 11.0; width of head including eyes: ♂ 9.5, ♀ 9.0; pronotum width (including pronotal collar): ♂ 10.0, ♀ 11.0; mesonotum width: ♂ 9.0, ♀ 10.0.

##### Biology.

Unknown.

##### Distribution.

China (Guangxi), Vietnam.

##### Remarks.

This species is similar to *Nipponosemia terminalis* but can be distinguished from the latter by a larger body size, the wings with large brown markings, the pronotum without markings, and the mesonotum with only one pair of obconical marks, in addition to the differences in male genitalia (Chou et al. 1993, 1997).

#### Nipponosemia virescens

Kato, 1926

http://species-id.net/wiki/Nipponosemia_virescens

Nipponosemia virescens Kato, 1926: 172; Hayashi 1979: 262; Duffels and van der Laan 1985: 164; Chou et al. 1997: 125; Lee and Hayashi 2004: 62.

##### Material examined.

No specimen available.

##### Biology.

This species is found in lowlands. Adults appear from April to July and both sexes are attracted to electric light at night (Lee and Hayashi 2004).

##### Distribution.

China (Taiwan).

##### Remarks.

Chou et al. (1997) incorrectly recorded this species from Japan. Considering the morphological similarity between this species and *Nipponosemia terminalis*,Lee and Hayashi (2004) suggested that they may represent one species and *Nipponosemia virescens* should be a kind of geographical variation within *Nipponosemia terminalis*, and the identity of this species needs to be confirmed when material becomes available. We included this species in the key in this paper according to the photograph provided by Lee and Hayashi (2004).

#### Nipponosemia longidactyla sp. n.

urn:lsid:zoobank.org:act:E7A095F0-914F-4F56-A6EA-FDBB2E4155EE

http://species-id.net/wiki/Nipponosemia_longidactyla

Figures 10 12

##### Material examined.

**Type material. Holotype:** ♂ (NWAF), **China**: Hainan Prov., Jianfengling Nature Reserve, 1-VI-1982, coll. Liu Yuanfu. **Paratypes:** 1♂ (NWAF), **China**: Hainan Prov., Limushan Nature Reserve, light trap, 26-V-1984, coll. Gu Maobin; 1♀ (NWAF), **China**: Hainan Prov., Jianfengling Nature Reserve, 980 m, light trap, 5-V-2008, coll. Fu Qiang. **Additional material.** 1♂ (NWAF), **China**: Hainan Prov., Jianfengling Nature Reserve, 960 m, light trap, 29-V-2011, coll. Yang Mingsheng.

##### Diagnosis.

This new species can be easily distinguished from other species of *Nipponosemia* by the following features: upper lobe of pygofer very long and arched, protruding inward; median lobe of uncus weak, with apex slightly produced, forming a small process curved upwards in ventral view; median clasper process well developed and twisted subbasally, forming a large process curved laterally; lateral clasper lobe roundly developed inwards and partially overlapped by the large subapical process of the median clasper process. In addition, we include one male specimen from Jianfengling Nature Reserve as additional material for this species based on its external morphology and the morphology of genitalia except for the aedeagus, as the aedeagus was broken, and its identity needs to be investigated further when more specimens become available.

##### Description.

**Figure 10.**
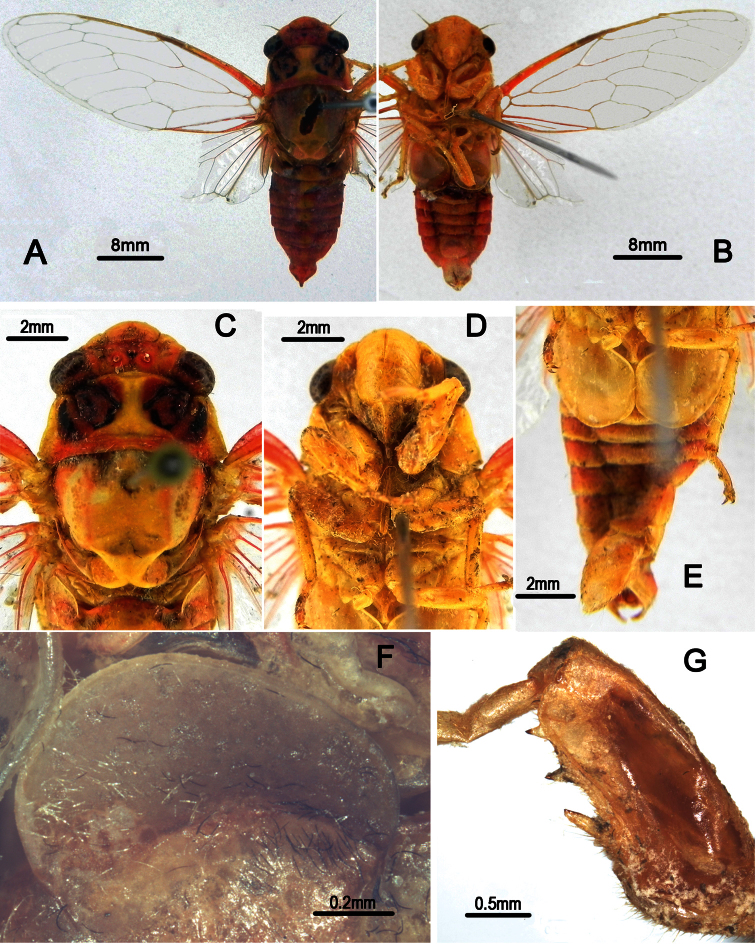
*Nipponosemia longidactyla* sp. n.,male. **A** habitus (holotype), dorsal view **B** habitus (holotype), ventral view **C** head and thorax (paratype), dorsal view **D** face (paratype) **E** abdomen and posterior part of thorax (paratype), ventral view **F** timbal and timbal cover (paratype), dorsal view **G** left fore leg (paratype), showing the spines on fore femur.

Head (Fig. 10A–B) mostly pale yellow, with reddish markings on vertex; clypeus yellow and depressed; ocellus orange, eye castaneous, distance between lateral ocellus and corresponding eye about as long as distance between lateral ocelli; face and gena yellow; rostrum yellowish with apical half light brown, extending to apex of mid coxae.

Pronotum (Fig. 10C) with central longitudinal yellowish fascia well broadened at both anterior and posterior parts; symmetrically with two large reddish brown to dark brown patches with border black; pronotal collar mostly reddish with lateral part pale yellow. Mesonotum (Fig. 10C) mostly yellowish, symmetrically tinged with red to reddish brown laterally in male but with blackish fasciae in female; cruciform elevation yellowish. Ventral surface of thorax mostly yellow, without distinct markings.

Legs (Fig. 10G) yellow except for reddish brown pretarsal claws; fore femur with primary spine long, digitate and slanted; secondary spine short, sharp and erect; subapical spine short, sharp and slanted.

Wings (Fig. 10A–B) hyaline, without any markings; veins in basal half reddish and yellowish apically.

Male abdomen (Fig. 10A–B) obconical, mostly dark red, with discontinuous central longitudinal yellowish fascia in dorsal view; timbal cover (Fig. 10F) ochreous; operculum (Fig. 10E) pale yellowish, obliquely ellipsoidal, subapical portion enlarged toward body center, extending slightly beyond posterior margin of abdominal sternite II, medial margins almost touching (holotype) or even touching (male paratype) each other. Female abdomen mostly black dorsally and yellowish brown ventrally, with discontinuous central longitudinal yellowish fascia in dorsal view, with reddish brown band on each posterior margin of terga 2–7; operculum small, semicircular, extending slightly beyond posterior margin of abdominal sternite II, both opercula well separated from each other.

**Figure 11.**
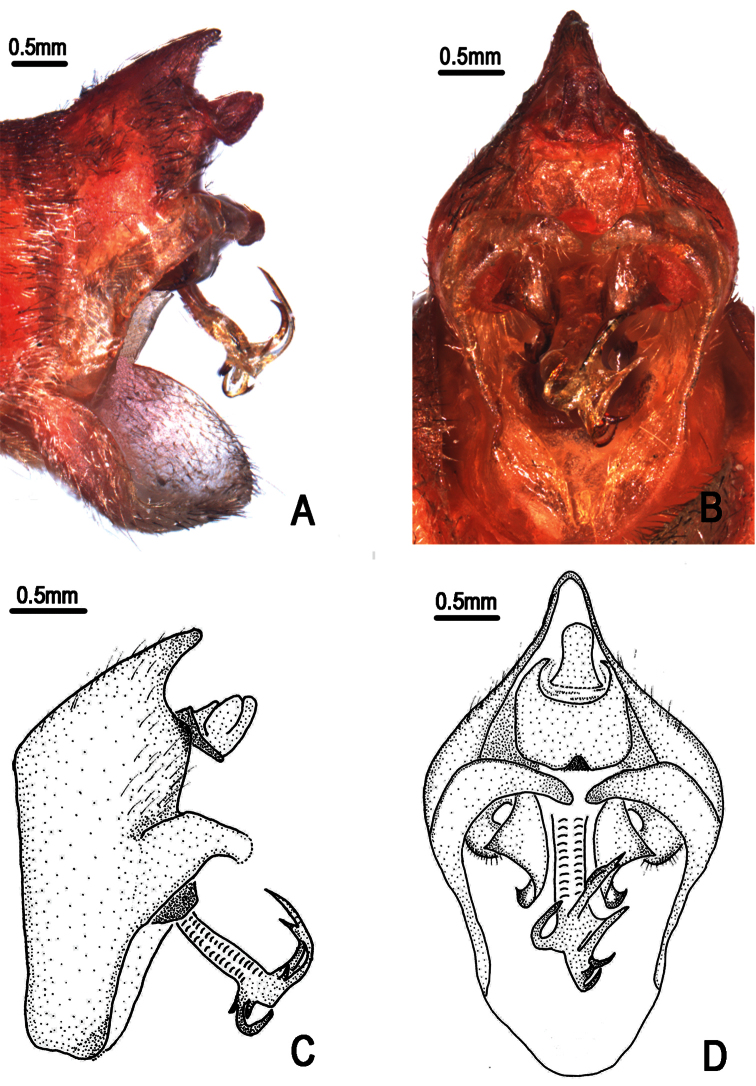
*Nipponosemia longidactyla* sp. n., male (holotype). **A, C** male genitalia, left lateral view **B, D** male genitalia, ventral view

Male genitalia (Fig. 11A–D). Pygofer oval in ventral view; dorsal beak long with obtuse tip; distal shoulder broadly convex in lateral view; upper lobe of pygofer remarkably long, digitate, curved inwardly. Uncus undeveloped in lateral view; apex of median lobe slightly produced, forming a small process curved upwards in ventral view. Clasper in ventral view with median clasper process well developed and twisted subbasally, forming a large process curved laterally; apex of median clasper process curved laterally and acute apically; lateral clasper lobe roundly developed inwards, partially overlapped by the large subapical process of median clasper process. Aedeagus with seven short to long processes apically and subapically, which are all pointed upward in ventral view but arranged into two groups in lateral view (three located ventrally and four dorsally). Posterior margin of sternite VII rounded.

**Figure 12.**
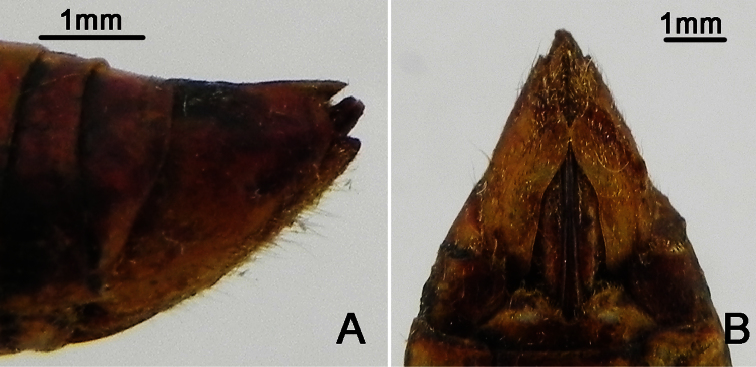
*Nipponosemia longidactyla* sp. n., female (paratype). **A** female genitalia, left lateral view **B **female genitalia, ventral view.

Female pygofer (Fig. 12A–B) with dorsal beak short and acute; ovipositor short, not extending beyond the end of abdomen; posterior margin of sternite VII with median incision very broad and relatively shallow, deep to about 1/2 the length of sternite VII.

**Measurement** (2♂♂, 1♀) (in mm). Length of body: ♂20.0–21.0, ♀ 21.0; length of fore wing: ♂21.0, ♀ 25.5; width of fore wing: ♂7.0, ♀ 8.0; width of head including eyes: ♂6.0, ♀ 6.5; width of pronotum (including pronotal collar):♂ 7.0, ♀ 7.5; width of mesonotum:♂ 6.5, ♀ 7.0.

##### Biology.

All the examined materials including the additional material were collected from the same tropical rainforest.

##### Distribution.

China (Hainan).

##### Etymology.

The specific name is derived from Latin prefix “*longi-*”and “*dactyla*” which refer to the long upper lobe of pygofer.

## Discussion

We review the cicada genus *Nipponosemia* and describe a new species, *Nipponosemia longidactyla* sp. n., for this genus in this paper. However, compared to other species of *Nipponosemia*, this new species has several peculiar characters, viz, the well developed upper lobe of pygofer and the complicated and twisted median clasper process, as indicate that this species might not be congeneric to other species of *Nipponosemia*. We tentatively place this new species in *Nipponosemia* until its status being addressed definitely via phylogenetic analysis.

Chen et al. (2012) reviewed the *Mogannia* from China and noted that the Cicadatrini (represented by *Mogannia*) could be a member of the subfamily Cicadettinae, but they tentatively retained it in the subfamily Cicadinae since other genera of Cicadatrini such as *Nipponosemia*, *Cicadatra*, *etc*. were not addressed. Herein, we scored the morphological attributes for *Nipponosemia* that are identified in Moulds (2005, fig. 59 and associated text) as defining subfamilies. Similar to *Mogannia*, *Nipponosemia* also appears to be more allied to the Cicadettinae in having the following morphological characters: 1) width of first cubital cell of hind wing at distal end much broader than second cubital cell (twice or more); 2) upper lobe of pygofer present; 3) large claspers dominating the whole 10^th^ abdominal segment; 4) uncus short, not dominant; 5) aedeagus restrained by claspers; and 6) fore wing vein CuA_1_ divided by crossvein (m-cu) so that proximal portion is shorter. However, we tentatively retained *Nipponosemia* in Cicadinae, as the phylogenetic relationship of genera in the Cicadatrini (*sensu* Lee and Hill 2010) with other related taxa needs to be adequately analyzed by more morphological features and molecular data from extensive sampling taxa.

**Figure 13.**
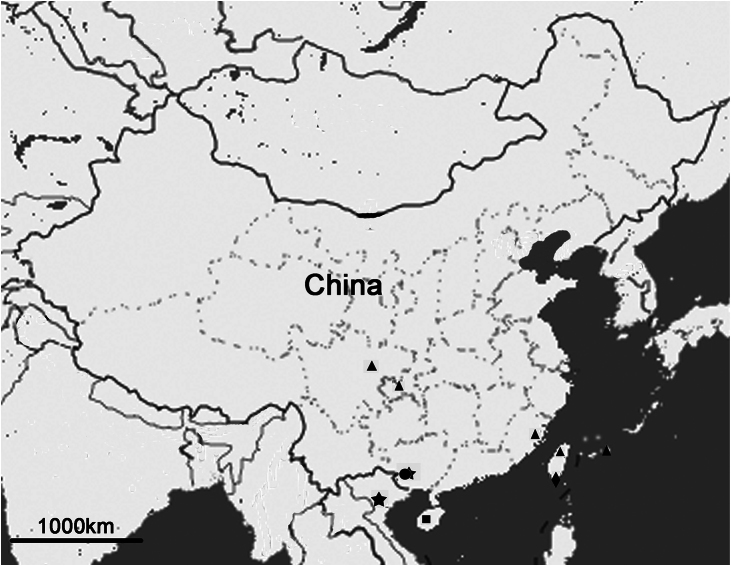
Distribution of *Nipponosemia* species. *Nipponosemia terminalis* (triangle); *Nipponosemia metulata* (round); *Nipponosemia guangxiensis* (five-pointed star); *Nipponosemia virescens* (diamond); *Nipponosemia longidactyla* sp. n. (square).

Regarding the biogeography of *Nipponosemia*, *Nipponosemia terminalis* has the widest distribution range among the five *Nipponosemia* species (Fig. 13), i.e., from southwestern China (Sichuan and Chongqing) to Taiwan Island and the southern Ryukyus. This disjunctive distribution indicates that *Nipponosemia terminalis* had dispersed over oceanic barriers during the Ice Ages. The remaining species of *Nipponosemia* are all restricted to narrow regions: *Nipponosemia guangxiensis* occurs in Guangxi Prov. of southern China and Vinh Phuc of northern Vietnam (Pham and Yang 2009); *Nipponosemia metulata* is only known from Guangxi Prov. of China (Chou et al. 1993, 1997); *Nipponosemia virescens* is restricted to southernmost Taiwan of China (Lee and Hayashi 2004); and *Nipponosemia longidactyla* sp. n. is currently only known from Hainan Island of China. The above distribution pattern indicates that this genus is distributed in the Oriental Region, particularly southern China and Pacific islands adjacent to the China Mainland. Higher biodiversity of this genus in the Oriental Region probably can be revealed when more biodiversity inventory projects covering biodiversity hotspots there are completed.

## Supplementary Material

XML Treatment for Nipponosemia

XML Treatment for Nipponosemia terminalis

XML Treatment for Nipponosemia metulata

XML Treatment for Nipponosemia guangxiensis

XML Treatment for Nipponosemia virescens

XML Treatment for Nipponosemia longidactyla
